# A Volume-Adjustable Artificial Womb for Extremely Preterm Infants

**DOI:** 10.3389/ti.2024.12947

**Published:** 2024-07-09

**Authors:** Jan Heyer, Franziska Schubert, Alexander L. Seitz, Yannick Steinle, Jutta Arens, Thorsten Orlikowsky, Ulrich Steinseifer, Thomas Schmitz-Rode, Sebastian V. Jansen, Mark Schoberer

**Affiliations:** ^1^ Department of Cardiovascular Engineering, Institute of Applied Medical Engineering, Helmholtz Institute, RWTH Aachen University and University Hospital, Aachen, Germany; ^2^ Engineering Organ Support Technologies Group, Department of Biomechanical Engineering, University of Twente, Enschede, Netherlands; ^3^ Pediatric Clinic, Neonatology Section, RWTH Aachen University and University Hospital, Aachen, Germany

**Keywords:** artificial womb, artificial amniotic sac, adjustable silicone sac, perinatal life support, extremely preterm infant

## Abstract

More than 13 million children are born preterm annually. Prematurity-related mortality accounts for 0.9 million deaths worldwide. The majority of those affected are Extremely Preterm Infants (gestational age less than 28 weeks). Immaturity causes organ failure and specific morbidities like germinal matrix hemorrhage, bronchopulmonary dysplasia, and necrotizing enterocolitis. Artificial womb and placenta technologies address these issues. As a bridge-to-life technology, they provide a liquid environment to allow organ maturation under more physiological conditions. The proposed artificial womb can adapt to fetal growth. Volume adjustment is achieved by removing fluid from the interspace between an inner and outer chamber. Results of the *in vitro* tests showed a temperature constancy of 36.8°C ± 0.3°C without pressure loss over 7 days. The volume of the inner sac was variable between 3.6 and 7.0 L. We designed a filtration and disinfection system for this particular purpose. This system has proven strong disinfection capabilities, effective filtering of metabolic waste, and the ability to avoid phospholipid washout. The presented artificial womb has sufficient volume variability to adapt to the physiologic growth of an extremely preterm neonate over a 4-week period. We regard this as an important step in the development of this bridge-to-life technology.

## Graphical Abstract

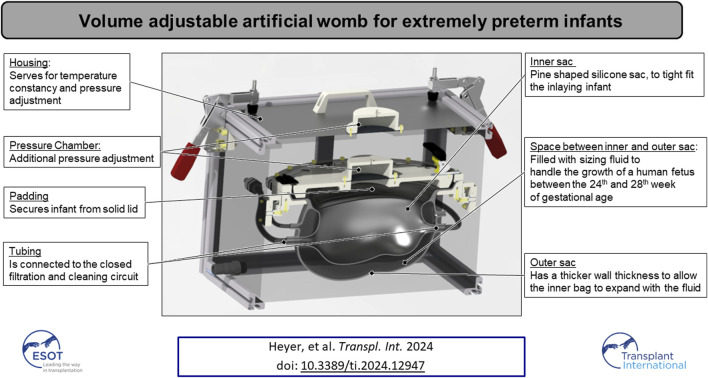

## Introduction

In 2020, more than 13 million children worldwide were estimated to be born preterm [gestational age (GA) less than 37 weeks], with more than 5% of mortality due to complications of preterm birth [1]. Neonatal intensive care serves to improve prematurity outcomes. Modern neonatal intensive care treatment uses a microenvironment to control respiratory gases, temperature, and humidity. Antenatal steroid prophylaxis, postnatal surfactant replacement therapy, and mechanical ventilation have enabled pulmonary gas exchange at the canalicular stage of lung development. This has allowed survival in the first place [2].

However, extremely small-for-gestational-age neonates (ELGANs, GA less than 28 weeks) have the highest therapy-related morbidity and mortality rates. Their vulnerability can be attributed to organ immaturity [3, 4], especially of the lungs and brain. Lung surfactant is detectable as early as 24 weeks of gestation [5], and is essential for the ability to breathe air as well as a prerequisite for functional lung development. Surfactant deficiency causes neonatal respiratory distress syndrome [5, 6]. The 23rd to 24th week of GA is currently the limit of viability, with survival rates below 10% at 23 weeks of gestation and 35% at 24 weeks of gestation [3]. Acute respiratory distress is the leading cause of mortality in this group [2]. Survival comes at the expense of lifelong structural and functional pulmonary morbidity [2].

Searching for a way out of this paradox, several research groups are developing artificial womb (AW) and artificial placenta (AP) technologies [7–10]. These technologies aim to overcome the current complications in therapy and establish a bridge-to-life approach. Recent achievements in animal models have demonstrated the potential of this technology [7, 11]. Patridge et al. and Usuda et al. were able to support lambs in their AW for 19–25 days [7], with a maximum of 28 days [11]. Fetal growth at mid-term was observed to be exponential [12]. The fetus almost doubles its weight between the 24th and the 28th weeks of GA [12, 13]. Furthermore, during the 24th week of GA, the multisensory and pain perception of the fetus [14] and the haptic system are at a decisive stage of development [15–17]. At this age, the fetus learns to discriminate between the environment and its own body through touch [15–17].

All animal studies published to date have required fixation of the animal in its surrounding bag in order to prevent movement and decannulation within the proposed artificial wombs [7]. Current AW Systems manage growth using an end-stage-size biobag where the fetus is held in position using manually adjusted magnets [7]. Such fixation of the fetus can have a serious impact on neurosensory and motor development. Furthermore, the artificial amniotic fluid is constantly drained of, flushing away the autologous surfactant in the fluid produced by the fetus [7–10]. This rather simple design of the currently used “biobags” has shown good functionality in the animal experimental setup [18] but has practical limitations for clinical use and does not cover the core needs of a developing fetus. The need for constant flushing with sterile fluids would be a practical limitation and an enormous logistic burden, even more so in environments that have a developing infrastructure. Keeping a closed, fluid-filled polymeric system sterile means a significant challenge in terms of filtration and disinfection technology. In addition to hygienic issues, the system should support neurodevelopment. Current biobags purposefully restrict fetal movement as described above. Moreover, biobags do not well simulate the elastic forces in the interaction between the developing fetal musculature and the uterus.

The AW approach presented here, the Liquid Filled Chamber (LFC), provides an adaptable volume that allows for fetal movement and growth without fixation. Furthermore, a closed-loop filtration system is added to prevent flushing of the autologous surfactant from the artificial amniotic fluid, as suggested by Bie et al. [18].

## Materials and Methods

### Requirements for the LFC

As a first step, the requirements for the LFC were identified by dividing the LFC into four functional units: the artificial amniotic sac (AAS), housing, amniotic fluid, and filtration system. The requirement specifications are shown in Table 1.

**TABLE 1:** Requirement specifications for LFC.

Unit	Requirement	Requirement specification	No.
AAS	Mimicking the physiological womb	A fluid environment for the AAS with materials that are elastic and can be shaped and sized to fit a fetus of the appropriate GA	R1
	Tight-fitting but not restrictive of physiological fetal movement	The AAS must be continuously or repeatedly adjustable relative to the size and growth of the neonate [12, 13, 19]	R2
	Growing system		R3
	Transfer and accessibility	Ability to open the AAS from outside the system for perinatal transfer as part of the EXIT procedure^a^, and for emergency interventions	R4
	Closed system	The AAS needs to generate a sealed ecosystem similar to the physiological womb	R5
	Umbilical cord interface	The AAS needs to incorporate a sealable interface for the throughput of the umbilical cord or an appropriate cannula	R6
Housing	Temperature control	The temperature needs to be physiological (37°C) and stable according to the body temperature	R7
	Pressure level	Pressure needs to be physiological and stable but adjustable according to the intrauterine pressure [20]	R8
Amniotic fluid	Mimicking the physiological fluid	Artificial amniotic fluid should have a similar composition to natural amniotic fluid	R9
	Leakage detection	Fluid leakage from or into the AAS should be detectable	R10
Filtering system	Excretion elimination	Toxic excretions need to be constantly filtered from the artificial amniotic fluid stream	R11
	Microorganism elimination	Infectious agents need to be eliminated to prevent sepsis [18]	R12
	Prevention of phospholipid washout	Phospholipid washout by the filtration system needs to be prevented [5, 6]	R13

^a^ EXIT-procedure: ex utero intrapartum treatment procedure.

Requirements R1, R2 and R3 result from the anatomy and growth dynamics of the neonate, and the maternal uterus. These are presented in Table 2 according to the literature values, with the standard deviation given in brackets. In the case of different values in the literature, the largest variation was chosen to obtain a conservative result. For intrauterine pressure, different values are reported in the literature [23–25]. For LFC, the minimum pressure at 24 weeks of GA was estimated to be 9.5 mmHg, according to Wiener et al. [25]. A peak pressure of 27.7 mmHg caused by uterine contraction was measured by Beard et al. [23], and this value was used as the upper pressure limit for the LFC.

**TABLE 2:** Parameters of the maternal womb and the neonate relative to GA.

GA in weeks	Crown to rump length (CRL) in mm [21, 22]	Chest circumference (CC) in mm [21]	Weight of the neonate in g [12]
22	195 (15)	161 (19)	477 (61)
23	204 (15)	170 (20)	568 (72)
24	213 (16)	180 (21)	670 (85)
25	233 (17)	189 (22)	785 (100)
26	232 (18)	199 (23)	913 (115)
27	242 (19)	209 (24)	1,055 (134)
28	250 (21)	217^a^ (25^a^)	1,210 (154)

^a^ Extrapolated from literature data.

### Design Targets for LFC

Design targets for the LFC System can be derived from the requirements defined in Table 1 and from the physiological parameters given in Table 2. The resulting design targets are listed in Table 3.

**TABLE 3:** Design targets for LFC.

Unit	No.	Design target
AAS	R1	The AAS is a fluid-filled system made of transparent silicone to ensure elasticity and to allow observation of the neonate. The shape and size were chosen with the values given in Table 2 plus two standard deviations to allow 95.5% of neonates to fit into the ASS
	R2	The AAS consists of a double-walled encasement (silicone). A sizing fluid is placed between the two walls. Passive or active removal of the sizing fluid enables volume adjustment. The sizing fluid system is isolated from the rest of the LFC
	R3	
	R4	The AAS features a lid that seals its inner system from the environment, but can be opened to have access to the neonate. The lid is designed to be atraumatic
	R5	The AAS is a closed system attached to the filtration system
	R6	The umbilical cord or its cannulas are routed outside the AAS for connection with the Artificial Placenta (AP)
Housing	R7 R8	The outer housing is a fluid-filled chamber that surrounds the AAS. The heat capacity of water is used to ensure a stable temperature of 37°C ± 1°C. The internal pressure can be adjusted between 10 mmHg and 30 mmHg
Amniotic fluid	R9	A commercially available medical-grade artificial amniotic fluid is chosen (Anmion Flush, Serumwerk Bernburg AG, Brenburg, Germany)
	R10	Leakage of different fluids is detectable in the system by photometric measurement
Filtration system	R11	The filtration system is designed as a closed circuit with different functional components: gross filtration, fine filtration (dialyzer), and disinfection (UV-light). The prevention of phospholipid washout in all systems is secured by an adjusted concentration gradient. The maximum flow rate of the filtration system is estimated to be 800 mL/min, resulting in the filtration of the entire fluid in 12.5 min
	R12	
	R13	

All parts of the LFC and its components were designed using CAD software (Autodesk Inventor, San Francisco, United States). Silicone parts were manufactured by hand (Silicone 620 A/B, Wacker Chemie AG, München, Germany). Special molding techniques were developed for each silicone component. Other parts were manufactured in the in-house mechanical workshop, and commercially available parts were integrated into the system.

### Verification Testing

The identified requirements (Table 1) were tested in individual setups. R1, R2 and R3 were tested in a volume adaptability test. R5, R7, R8, and R10 were tested in a 7-day sealing and stability test. R12 and R13 were tested in a filter test. R4 and R6 are determined by the selected lid construction and the respective sealing technique. No verification is required for the structural part. For R9 and R11, commercially available CE-marked medical products were used.

In the volume adaptability test, the principle of the sizing fluid system was tested. The AAS was filled using water as a surrogate for the artificial amniotic fluid according to the volumes given by the state at 24 weeks of GA. Manikins representing the 3D reconstruction of the fetal body from MRI data were manufactured by our consortium partners from TU/e Eindhoven, van Haren et al. [26]. The fetal manikins were laid in the AAS to visualize the volume adjustability. At the beginning of the test, a manikin at 24 weeks of GA was used. After increasing the volume, the manikin was replaced by one mimicking 28 weeks of GA. This would represent the body size of a fetus at the expected end of the treatment period. The volume of adaptive sizing fluid between the AAS walls was removed stepwise in 500 mL increments, and the same amount of fluid was added to the AAS to mimic neonatal growth. After each fluid removal step, a photograph of the system was taken for visual evaluation. The steps were repeated until the complete removal of the sizing fluid. The internal “intrauterine” pressure of the AAS LFC was measured using two calibrated pressure sensors at the in- and outlet of the AAS (Xtrans, CODAN Companies, Rodan, Denmark).

For the sealing and operability test, the completely assembled LFC was tested for leakage, temperature stability, and AAS intrauterine pressure constancy for 7 days. We believe that 7 days is a representative time frame for the maximum therapy duration of 4 weeks in terms of leakage, temperature, and pressure constancy. Water was again used as a surrogate for the artificial amniotic fluid, and the volume flow was set at 800 mL/min, the maximum filter flow as depicted in Table 3. The umbilical cord interface was sealed with a closed silicone tube mimicking the umbilical cord.

Leakage detection was performed visually by a red coloring ((Reaktiv-Rot 120 (λ = 509 nm), Merck, Darmstadt, Germany) of the surrounding water inside the LFC housing, such that leakage into the AAS could be detected. At the beginning of the test, a 100 mL sample of the dyed surrounding water was kept as a positive reference and 5 mL of the water inside the AAS was taken as a negative reference. Every 24 h of the test, a 5 mL sample was taken from each circuit. Samples and references were analyzed with respect to absorption at λ = 509 nm (Ultrospec 2100 pro, Amersham Bioscience, Amersham, United Kingdom). Temperature was measured (Omgea, Deckenpfronn, Germany) inside the AAS, inside the housing, and inside the heating circuit as a reference. The sensors were calibrated in a heating bath at 37°C using an analog laboratory thermometer. The LFC was filled with fluid 12 h prior to the beginning of the test to ensure a homogeneous starting temperature of 22°C in all components. Temperature values were taken automatically every 5 minutes. Two-point calibrated pressure sensors (Xtrans, CODAN Companies, Rodan, Denmark) were placed at the top of the lid and at the in- and outlet of the inner AAS to measure the AAS “intra uterine” pressure of the system. Automatic recording of the pressure sensors every 60 min was performed using the institute’s data acquisition system.

The filter was tested for elimination of microorganissms and potential washout of phospholipids. In total, 500 mL of artificial amniotic fluid in contact with ambient air was incubated for 14 days at 37°C to allow microorganisms to grow within the fluid. From this fluid, three 50 mL samples (Falcon, Thermo Fisher Scientific, Waltham, Massachusetts, United States) were prepared. Two of these were exposed to the filtration system’s UV-C lamp for 5 min, and one was left untreated as a reference. All three samples were tested for microorganisms with a standard smear test. The washout of phospholipids through the dialyzer of the filtration system was tested in two separate experiments. Phospholipid EMPIGEN^®^ BB Detergent (Merck, Darmstadt, Germany) (271.44 DA) was used to mimic the autologous phospholipid Dipalmitoylphosphatidylcholine (735 DA) of the neonate on the AAS side. To prevent washout, a phospholipid 3-(N,N-Dimethylmyristylammonio)-propanesulfonate (Merck, Darmstadt, Germany) of similar size (391.65 Da) was used in the filtration system to eliminate a concentration gradient across the dialyzer membrane. For the test, both fluids (AAS side and filtration system side) were fully saturated using the respective phospholipids. The filtration system was run at 800 mL/min for a duration of 240 min. A reference test circuit was set up using fully saturated fluid on the AAS side and no phospholipids on the filtration side of the dialyzer. Repeated samples were collected between 0 and 360 min and subsequently analyzed using the bubble test proposed by Pattle et al. [27].

## Results

### Liquid Filled Chamber (LFC) Design

**FIGURE 1:**
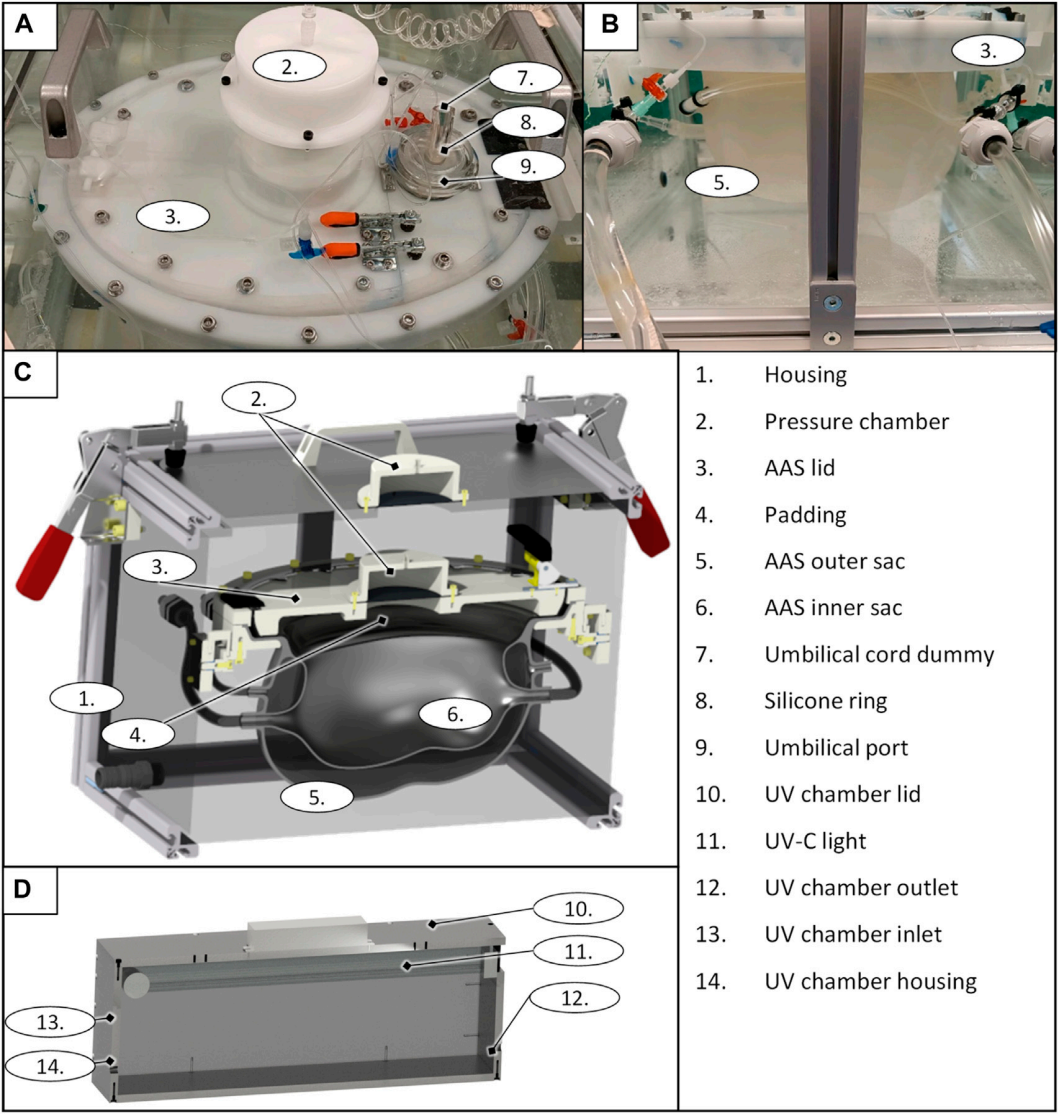
LFC prototype top view **(A)**; side view **(B)**, general design of the liquid-filled chamber as a CAD rendering sectional view **(D)**, and the UV chamber of the filtration system as a CAD rendering sectional view **(D)**.

The fully assembled LFC is shown in Figure 1. The housing (Figure 1 1) represents the outer subsystem of the LFC. The outer subsystem is intended to ensure a constant temperature at 37°C (R7) using a heating unit (Figure 1 5). The internal pressure (R8) on the AAS (Figure 1 6) is set by the geodetic pressure of the water level above the AAS and additionally by a pressure chamber, which increases the overall pressure in the housing unit if needed.

The AAS consists of an inner sac, an outer sac, and its lid (Figure 1 3). The sizing fluid (R2 & R3) is enclosed between the inner sac and outer sac of the AAS and can either be pushed out passively (due to growth) or externally adjusted. Both sacs and the sizing fluid mimic the elasticity of the maternal womb (R1). The outer sac has a wall thickness of 3 mm–4 mm, and the inner sac has a wall thickness of 1.5 mm–2 mm (R2 & R3). The volume of the outer sac is 7.2 L and the volume of the initial inner sac is 3.6 L, leaving 3.6 L of volume for growth in the interspace between the two sacs. The shape of the initial inner sacs was defined along the CRL and CC (Table 2) as the axes of two perpendicular ellipses. The pine-shaped bottom of the two sacs (Figure 1) predefines the position of the neonate. The shape and growth–adaptive system provides enough space for the growing neonate, especially in the hip and head regions (R1). The AAS lid closes and seals the inner sac opening (R5), providing padding (Figure 1 4) on the inside to protect the neonate. The housing and the AAS lid have a built-in pressure chamber (Figure 1 2), consisting of an air-filled chamber and a flexible membrane connected to the housing system and the padding. Air can be compressed in this chamber to generate extra pressure on the corresponding system. The AAS lid further includes a port for the umbilical cord (Figure 1 9). The umbilical port acts as an interface between the inner sac and the outer system to allow the neonate’s umbilical cord or the cannulation tubing to pass to the artificial placenta (R6). The port seals the umbilical cord (Figure 1 7 illustrated with an umbilical cord dummy) or the cannulation tubing using a pressurized flexible silicone ring (Figure 1 8).

The filtration system is attached to the inner sac and prevents contamination of the neonate’s environment within the LFC. It comprises a roller pump (HL 20, Marquet, Raststatt, Germany), a changeable gross filter (0.5 mm–0.7 mm), a UV–C disinfection unit, and a hemodialyzer (High Flux FX 50, Fresenius medical care, Bad Homburg, Germany). The roller pump pumps the artificial amniotic fluid from the AAS system through the filtration system. The gross filter removes skin and hair particles. The UV–C unit uses a UV–C lamp (Osram UV-Lamp HNS-L 95W, Osram, Garching, Germany) (Figure 1 11) assembled in a PMMA box (135 mm × 580 mm × 176 mm) (Figure 1 10 and 14) with UV sublimated coating (Clear UVC, Solarscreen, Foetz, Luxembourg). It additionally serves as a reservoir for the AAS circuit. The UV-C lamp is automatically switched off for 15 min every 30 min to prevent overheating (R12). If changes in the water level exceed ±1 L (e.g., due to leakage), the pump and UV lamp are automatically switched off, and an acoustic alarm is triggered. The hemodialyzer removes harmful excretions from the artificial amniotic fluid (R11) using a conventional dialysate with added phospholipids (EMPIGEN^®^ BB Detergent, Merck, Darmstadt, Germany) to prevent washout of the neonate’s autologous phospholipids (R13).

### *In Vitro* Verification of LFC

**FIGURE 2:**
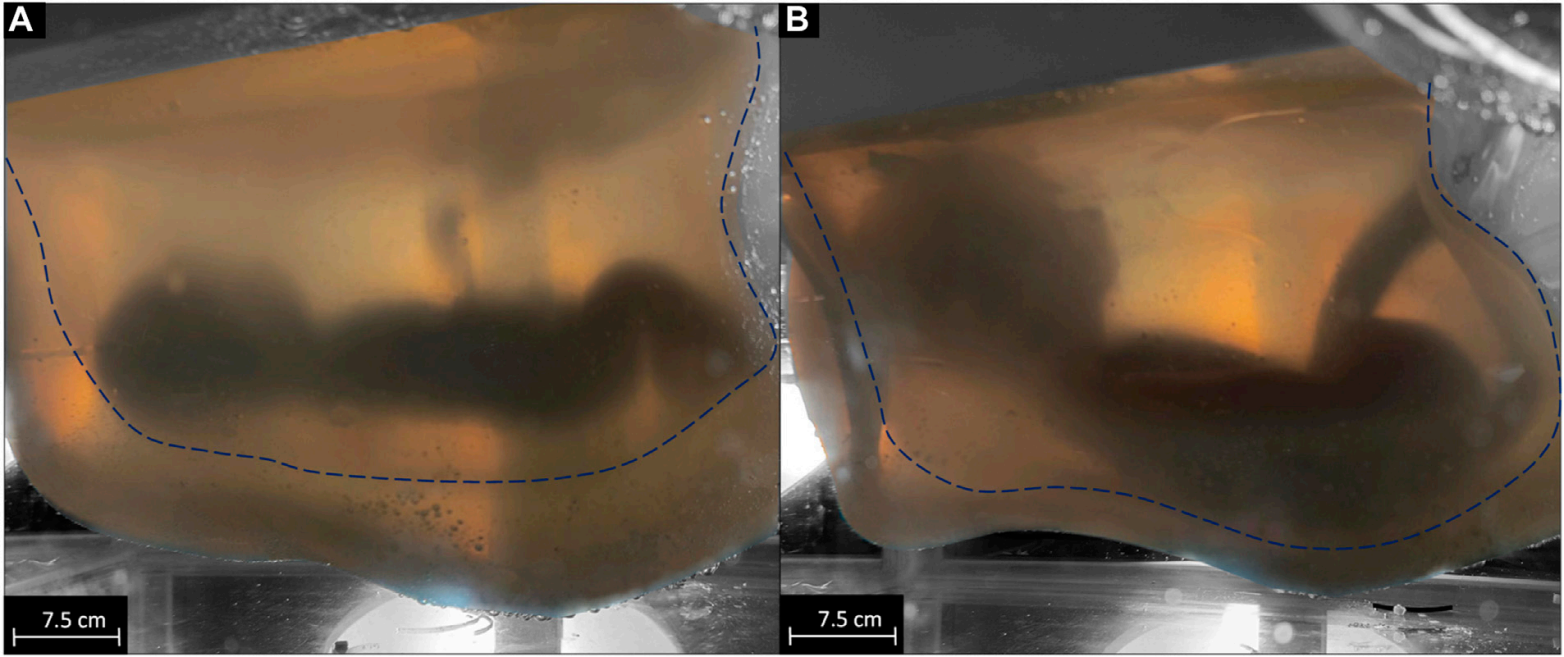
visualization of the growth of the system from 3.6 L filling volume of the inner sac [dashed line **(A)**] to 6.8 L filling volume of the inner sac [dashed line **(B)**].

The results of the volume adaptability test of the system to compensate for the growth of the neonate are shown in Figure 2. A total of 3.4 L of sizing fluid was manually removed from the initial state (Figure 2A) to the maximum expanded state of the AAS (Figure 2B). It was not possible to extract the full 3.6 L of sizing fluid, so 0.2 L remained in the interspace between the sacs. Falling of the folds leads to an inversion of the outer sac. As the volume increased, the AAS pressure increased linearly from 8 to 21 mmHg.

The seal and operability test results are shown in Table 4. The Absorption, Temperature, and Pressure parameters are listed according to their measurement location for each day starting after the heating period, which took 5 h.

**TABLE 4:** Resulting absorption rates, temperature and pressure data from the long-term sealing and operational stability test, and standard deviations are given in brackets.

Days		1	2	3	4	5	6	7
Absorption	*ε*_outer_	0.303	0.289	0.304	0.299	0.308	0.308	0.293
	*ε*_inner_	0.003	0.024	0.015	0.010	0.011	0.010	0.012
Temperature	T_inner_	37.37 (0.27)	36.88 (0.33)	36.66 (0.05)	36.69 (0.05)	36.69 (0.07)	36.71 (0.07)	36.71 (0.06)
	T_outer_	39.24 (0.54)	38.51 (0.12)	37.55 (0.14)	37.30 (0.09)	37.57 (0.09)	37.87 (0.48)	37.46 (0.40)
	T_reference_	39.85 (0.50)	39.36 (0.08)	39.43 (0.05)	39.41 (0.04)	39.43 (0.05)	39.43 (0.05)	39.41 (0.04)
Pressure	p_padding_	27.80 (0.22)	27.61 (0.15)	27.03 (0.40	27.00 (0.10)	26.51 (0.25)	26.60 (0.15)	26.65 (0.23)
	p_inner_in_	30.35 (0.48)	30.17 (0.32)	29.56 (0.08)	29.09 (0.10)	28.86 (0.05)	29.04 (0.19)	29.29 (0.05)
	p_inner_out_	28.72 (0.38)	28.50 (0.51)	29.28 (0.16)	28.81 (0.06)	28.54 (0.09)	28.60 (0.21)	28.26 (0.22)

**FIGURE 3:**
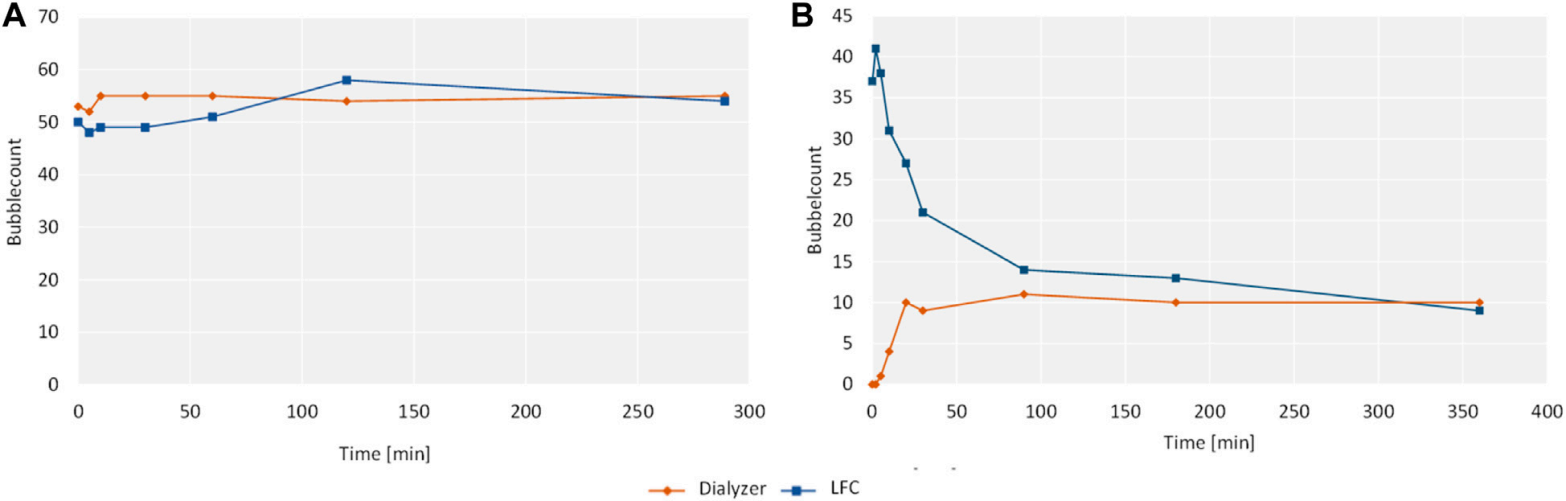
Results of the filtration system washout with bubble count on the y-axis and time on the x-axis.

In the filter test, the standard smear showed a negative result for both samples without any detectable growth of microorganisms, in contrast to the reference sample, which showed a positive result and the formation of a bacterial lawn. Results for the phospholipid washout are given in Figure 3. With an equal concentration of phospholipids of the same size on both sides of the hemodialyzer membrane, no change in the phospholipid concentration could be observed (Figure 3A). In the negative reference without adjusted phospholipid concentration in the dialysate, a washout of phospholipids was detected with an apparent decrease in total phospholipid concentration (Figure 3B).

## Discussion

The size and shape of the AAS were designed to appropriately fit a human neonate (at the 24th week of GA) according to growth data reported in the literature (R1). The elasticity of the double-walled AAS provides a gentle resistance to the movements of the neonate (R2). Published data on uterine compliance or uterine muscle E-module were not sufficient for our purpose. Theoretically, the compliance of the AAS can be adjusted by the thickness of its wall or by choosing a different medical grade silicone. The AAS allows the interaction between fetal movements and an elastic but still restrictive surrounding just like *in utero*. This interaction makes it possible to develop the necessary steps in the haptic sense and in neuro-muscular and musculoskeletal development [28]. The lid of the AAS is designed to enable the two different transfer procedures (R4) that are currently the focus of our team. Furthermore, the lid provides access (R4) to the neonate, e.g., for emergency procedures.

The ability of the AAS to compensate for neonatal growth (R1, R2, R3) was demonstrated by a 3.4 L (94%) increase in the inner sac volume. According to the data given in Table 2, the resulting volume adjustability should fit 90% of all neonates from GA 24 to GA 28. The AAS intrauterine pressure can be manually adjusted from 0 mmHg to 30 mmHg (R8). External adjustment is possible via water level variations in the housing. Internal adjustment can be achieved through the pressure chamber. Increasing pressure due to the growth of the neonate can be compensated by the same mechanisms, enabling the AAS to maintain a constant pressure for the neonate.

The long-term operability of the LFC System was verified in a 7-day test. No leakage could be detected. The variation in the absorption values of the colored liquid was within the measurement accuracy of the UltroSpec 2100. Temperature and pressure values were found to be constant over time and within the predefined range (R5, R6, R7, R8).

For the filtration system, a commercially available CE-marked hemodialyzer (R11) was used so that the elimination of metabolic waste in the amniotic fluid could be assumed. The elimination of microorganisms using the UV disinfection unit proved successful in the static test. Exposure of the fluid samples to UV light for 5 min was sufficient to disinfect the sample. Given the current volume of the UV-irradiated reservoir (6.2 L) and the intended flow rate of the filtration system of 800 mL/min, this results in an average passage time through the UV-irradiated reservoir of 7.75 min, giving sufficient time for disinfection (R12).

A stable level of phospholipids by harmonizing the concentration gradient on the dialysate side of the hemodialyzer showed no washout of the phospholipids within the AAS. However, it is still possible that an exchange of phospholipids across the dialyzer membrane takes place. Although it would be favorable to keep the autologous phospholipids completely within the AAS circuit, a mixture of autologous and artificial phospholipids is not expected to be harmful to the neonate. However, this needs to be further investigated.

All LFC surfaces that form the inner fluid circuit are made of sterilizable materials. Therefore, a sterile starting point can be achieved with the system. A static test for sterility has been presented in this work. Both conditions lead to the assumption of complete operational sterility. Nevertheless, this must be proven in a further study during the operation of the prototype.

Current artificial womb systems tested in animal models by other researchers are designed to place the neonate on its side or back and compensate for growth by adjusting magnets on the biobag. Our novel design lets neonates float in our system without contact with a potentially harmful solid floor. The buoyancy generated by the different fluid-filled sacs and the surroundings, in combination with the potential for neonatal movement within the system, is an evolution toward a more physiologic artificial womb. This is ensured by the inner shape and the tight-fitting elasticity of the inner chamber together with the neonate being in a state of buoyancy. We expect that this will positively influence the naturalness of movements and thus motor development. However, the movements are also restricted by the tight fit and shape of the inner sac analogous to the physiological womb. A definitive statement on the possibilities of fetal movement will have to be made with an LFC prototype adapted in shape and elasticity to the chosen animal model in future tests.

It is an inherent characteristic of artificial womb technology that it creates an artificial barrier to sensory contact between neonates and parents. This is the most substantial contrast to the dyadic relationship between mother and child. The importance of early bonding through skin-to-skin contact between mother and child for attachment in later life has been well examined [29]. Kangaroo-Mother-Care has been proven to reduce preterm morbidity and mortality [30].

Introducing delivery room skin-to-skin care into ELGANs has proven to improve subsequent mother-infant interactions and reduce the expression of stress-response genes [31, 32]. Current WHO recommendations for the care of preterm or low birth weight infants strongly advocate early and continuous Kangaroo mother care (KMC) [33]. We acknowledge the potential of artificial womb technology to negatively impact parent-child attachment. It is essential to develop procedures and techniques to reduce this risk. Technical solutions aimed at supporting visual and auditory contact could be well imagined: a clearer picture of the child could be transmitted through a camera. Acoustic signals like speech and physiological noises from the mother, like a heartbeat, could be transmitted by appropriate techniques. Furthermore, removing the outer housing would allow for closer sensory contact between parents and neonates. Therefore, additional concepts must be developed to maintain the pressure adaptability, temperature stability and buoyancy generated by the outer chamber.

## Conclusion

This study demonstrates the *in vitro* functionality of the volume-adjustable artificial womb. The system was verified to be adjustable in volume and pressure. It was temperature stable during a 7-day operation. The size of the system fits the size of the target patient group. It was demonstrated that phospholipid washout can be prevented within a closed-circuit disinfection and filtration system. In addition, the successful elimination of metabolic waste products and the effective antiseptic treatment of amniotic fluid were proven in a static *in vitro* test and mathematically transferred to the dynamic system. We have presented an artificial uterus that allows neonatal growth and movement. This is an important step in the transition to artificial womb technology as a bridge-to-life technology for the smallest of patients.

## Data Availability

The original contributions presented in the study are included in the article/Supplementary Material, further inquiries can be directed to the corresponding author.

## References

[1] Ohuma EO Moller A-B Bradley E Chakwera S Hussain-Alkhateeb L Lewin A National, Regional, and Global Estimates of Preterm Birth in 2020, With Trends From 2010: A Systematic Analysis. Lancet (London, England) (2023) 402(10409):1261–71. 10.1016/S0140-6736(23)00878-4 37805217

[2] Jobe AH Bancalari E. Bronchopulmonary Dysplasia. Am J Respir Crit Care Med (2001) 163(7):1723–9. 10.1164/ajrccm.163.7.2011060 11401896

[3] Stoll BJ Hansen NI Bell EF Walsh MC Carlo WA Shankaran S Trends in Care Practices, Morbidity, and Mortality of Extremely Preterm Neonates, 1993-2012. JAMA (2015) 314(10):1039–51. 10.1001/jama.2015.10244 26348753 PMC4787615

[4] Stoll BJ Hansen NI Bell EF Shankaran S Laptook AR Walsh MC Neonatal Outcomes of Extremely Preterm Infants From the NICHD Neonatal Research Network. Pediatrics (2010) 126(3):443–56. 10.1542/peds.2009-2959 20732945 PMC2982806

[5] Joshi S Kotecha S. Lung Growth and Development. Early Hum Dev (2007) 83(12):789–94. 10.1016/j.earlhumdev.2007.09.007 17905543

[6] Gupta A Paria A. Transition From Fetus to Neonate. Surgery (Oxford) (2016) 34(12):593–6. 10.1016/j.mpsur.2016.10.001

[7] Partridge EA Davey MG Hornick MA McGovern PE Mejaddam AY Vrecenak JD An Extra-Uterine System to Physiologically Support the Extreme Premature Lamb. Nat Commun (2017) 8:15112. 10.1038/ncomms15112 28440792 PMC5414058

[8] Gray BW El-Sabbagh A Zakem SJ Koch KL Rojas-Pena A Owens GE Development of an Artificial Placenta V: 70 H Veno-Venous Extracorporeal Life Support After Ventilatory Failure in Premature Lambs. J Pediatr Surg (2013) 48(1):145–53. 10.1016/j.jpedsurg.2012.10.030 23331807 PMC4076781

[9] Miura Y Saito M Usuda H Woodward E Rittenschober-Böhm J Kannan PS *Ex-Vivo* Uterine Environment (EVE) Therapy Induced Limited Fetal Inflammation in a Premature Lamb Model. PloS one (2015) 10(10):e0140701. 10.1371/journal.pone.0140701 26473607 PMC4608829

[10] van der Hout-van der Jagt MB Verweij EJT Andriessen P de Boode WP Bos AF Delbressine FLM Interprofessional Consensus Regarding Design Requirements for Liquid-Based Perinatal Life Support (PLS) Technology. Front Pediatr (2021) 9:793531. 10.3389/fped.2021.793531 35127593 PMC8809135

[11] Miura Y Usuda H Watanabe S Woodward E Saito M Musk GC Stable Control of Physiological Parameters, But Not Infection, in Preterm Lambs Maintained on *Ex Vivo* Uterine Environment Therapy. Artif organs (2017) 41(10):959–68. 10.1111/aor.12974 28891072

[12] Hadlock FP Harrist RB Martinez-Poyer J. In Utero Analysis of Fetal Growth: A Sonographic Weight Standard. Radiology (1991) 181(1):129–33. 10.1148/radiology.181.1.1887021 1887021

[13] Gardosi J Mongelli M Wilcox M Chang A. An Adjustable Fetal Weight Standard. Ultrasound Obstet Gynecol (1995) 6(3):168–74. 10.1046/j.1469-0705.1995.06030168.x 8521065

[14] Simons SHP Tibboel D. Pain Perception Development and Maturation. Semin Fetal neonatal Med (2006) 11(4):227–31. 10.1016/j.siny.2006.02.010 16621747

[15] Mueller SM Winkelmann C Grunwald M. Human Touch in Healthcare. Berlin, Heidelberg: Springer Berlin Heidelberg (2023).

[16] Grunwald M. editor. Human Haptic Perception: Basics and Applications. Basel: Birkhäuser Basel (2008).

[17] Andersen PA. Haptic Perception in the Human Foetus. In: Grunwald M, editor. Human Haptic Perception: Basics and Applications. Basel: Birkhäuser Basel (2008).

[18] de Bie FR Davey MG Larson AC Deprest J Flake AW. Artificial Placenta and Womb Technology: Past, Current, and Future Challenges Towards Clinical Translation. Prenatal Diagn (2021) 41(1):145–58. 10.1002/pd.5821 32875581

[19] Moore KL Persaud TVN Torchia MG. The Developing Human: Clinically Oriented Embryology. 10th ed. Philadelphia, PA: Elsevier (2016).

[20] Sideris IG Nicolaides KH. Amniotic Fluid Pressure During Pregnancy. Fetal Diagn Ther (1990) 5(2):104–8. 10.1159/000263555 2130828

[21] Chambers HM Knowles S Staples A Tamblyn M Haan EA. Anthropometric Measurements in the Second Trimester Fetus. Early Hum Dev (1993) 33(1):45–59. 10.1016/0378-3782(93)90172-Q 8319554

[22] Martinovic J. editor. Practical Manual of Fetal Pathology. 1st ed. Cham: Springer International Publishing Imprint Springer (2021).

[23] Beard RW Nathanielsz PW. Fetal Physiology, and Medicine: The Basis of Perinatology, 2nd Edition. In: Dekker M, editor. Reproductive Medicine, Vol.6. New York, Basel, London: Butterworths (1984).

[24] Csapo A. The Diagnostic Significance of the Intrauterine Pressure. I. Obstetrical Gynecol Surv (1970) 25(5):403–35. 10.1097/00006254-197005000-00001 4913108

[25] Weiner CP Heilskov J Pelzer G Grant S Wenstrom K Williamson RA. Normal Values for Human Umbilical Venous and Amniotic Fluid Pressures and Their Alteration by Fetal Disease. Am J Obstet Gynecol (1989) 161(3):714–7. 10.1016/0002-9378(89)90387-6 2675602

[26] van Haren JS van der Hout-van der Jagt MB Meijer N Monincx M Delbressine FLM Griffith XLG Simulation-Based Development: Shaping Clinical Procedures for Extra-Uterine Life Support Technology. Adv Simul. 10.1186/s41077-023-00267-y PMC10693037 38042828

[27] Pattle RE Kratzing CC Parkinson CE Graves L Robertson RD Robards GJ Maturity of Fetal Lungs Tested by Production of Stable Microbubbles in Amniotic Fluid. Br J Obstet Gynaecol (1979) 86(8):615–22. 10.1111/j.1471-0528.1979.tb10823.x 583024

[28] Verbruggen SW Loo JHW Hayat TTA Hajnal JV Rutherford MA Phillips ATM Modeling the Biomechanics of Fetal Movements. Biomech Model mechanobiology (2016) 15(4):995–1004. 10.1007/s10237-015-0738-1 PMC4945693 26534772

[29] Tessier R Cristo M Velez S Giron M de Calume ZF Ruiz-Palaez JG Kangaroo Mother Care and the Bonding Hypothesis. Pediatrics (1998) 102(2):e17. 10.1542/peds.102.2.e17 9685462

[30] Conde-Agudelo A Díaz-Rossello JL. Kangaroo Mother Care to Reduce Morbidity and Mortality in Low Birthweight Infants. Cochrane database Syst Rev (2016) 2016(8):CD002771. 10.1002/14651858.CD002771.pub4 27552521 PMC6464509

[31] Hucklenbruch-Rother E Vohlen C Mehdiani N Keller T Roth B Kribs A Delivery Room Skin-To-Skin Contact in Preterm Infants Affects Long-Term Expression of Stress Response Genes. Psychoneuroendocrinology (2020) 122:104883. 10.1016/j.psyneuen.2020.104883 33027708

[32] Mehler K Hucklenbruch-Rother E Trautmann-Villalba P Becker I Roth B Kribs A Delivery Room Skin-To-Skin Contact for Preterm Infants-A Randomized Clinical Trial. Acta Paediatr (Oslo, Norway 1992) (2020) 109(3):518–26. 10.1111/apa.14975 31423649

[33] World Healt Organization. WHO Recommendations for Care of the Preterm or Low Birth Weight Infant (2022). 36449655

